# Runx2-Modified Adipose-Derived Stem Cells Promote Tendon Graft Integration in Anterior Cruciate Ligament Reconstruction

**DOI:** 10.1038/srep19073

**Published:** 2016-01-08

**Authors:** Xin Zhang, Yong Ma, Xin Fu, Qiang Liu, Zhenxing Shao, Linghui Dai, Yanbin Pi, Xiaoqing Hu, Jiying Zhang, Xiaoning Duan, Wenqing Chen, Ping Chen, Chunyan Zhou, Yingfang Ao

**Affiliations:** 1 Institute of Sports Medicine, Beijing Key Laboratory of Sports Injuries, Peking University Third Hospital, Beijing, China; 2 Department of Biochemistry and Molecular Biology, Peking University School of Basic Medical Sciences, Beijing, China

## Abstract

Runx2 is a powerful osteo-inductive factor and adipose-derived stem cells (ADSCs) are multipotent. However, it is unknown whether Runx2-overexpressing ADSCs (Runx2-ADSCs) could promote anterior cruciate ligament (ACL) reconstruction. We evaluated the effect of Runx2-ADSCs on ACL reconstruction *in vitro* and *in vivo*. mRNA expressions of osteocalcin (OCN), bone sialoprotein (BSP) and collagen I (COLI) increased over time in Runx2-ADSCs. Runx2 overexpression inhibited LPL and PPARγ mRNA expressions. Runx2 induced alkaline phosphatase activity markedly. In nude mice injected with Runx2-ADSCs, promoted bone formation was detected by X-rays 8 weeks after injection. The healing of tendon-to-bone in a rabbit model of ACL reconstruction treated with Runx2-ADSCs, fibrin glue only and an RNAi targeting Runx2, was evaluated with CT 3D reconstruction, histological analysis and biomechanical methods. CT showed a greater degree of new bone formation around the bone tunnel in the group treated with Runx2-ADSCs compared with the fibrin glue group and RNAi Runx2 group. Histology showed that treatment with Runx2-ADSCs led to a rapid and significant increase at the tendon-to-bone compared with the control groups. Biomechanical tests demonstrated higher tendon pullout strength in the Runx2-ADSCs group at early time points. The healing of the attachment in ACL reconstruction was enhanced by Runx2-ADSCs.

Successful anterior cruciate ligament (ACL) reconstruction using a tendon graft, such as semitendinosus and gracilis tendons, depends on the integration of the tendon with the bone. The integration of tendon grafts as replacements for the ACL is still barely satisfactory in about 13% of the cases. Delayed healing of the tendon-to-bone not only impairs the return to functional activities, but also causes postoperative anterior-posterior laxity and failure^1^. Improved tendon-to-bone healing is clinically significant, enabling earlier and more aggressive rehabilitation and return to work or sport.

The healing of tendon-to-bone includes the invasion of Sharpey fibers from the bone tunnel. The renewal of collagen fiber continuity between the tendon and bone induces an indirect type of insertion^2^. Normal insertion sites of the ACL are not the same as the re-established tendon-to-bone attachment. The reconstructed ACL attachment probably remains an abnormal structure for several months, despite early rehabilitation after surgery. In the early period of rehabilitation, a ‘bungee-effect’ or ‘windshield-wiper’ may be induced by undesirable tendon-to-bone healing^3^. Thus, it is critical to develop a method to accelerate tendon graft integration in the early phase of ACL reconstruction.

Adipose-derived stem cells (ADSCs), a type of adult mesenchymal stem cell, are capable of self-renewal and increasing proliferation. Compared with bone marrow-derived stem cells, ADSCs can be obtained in significant numbers from adipose tissues, without the limitations of age-related decline of bone marrow stem cells or the low frequency of osteoprogenitors in marrow. Bone regeneration using ADSCs has been reported recently^4^. Osteogenic differentiation of stem cells using gene transfection is an effective approach for bone regeneration.

Runx2 is a member of RUX family transcription factors, which contains a conserved motif of 128 amino acids, and is highly homologous to the Runt protein of Drosophila melanogaster. The *Runx2* gene was first cloned in 1997 and is exclusively expressed in bone tissue or osteoblasts^5^. Runx2 is a downstream transcriptional activator that regulates osteogenesis directly and plays a vital role in osteoblast differentiation during embryonic development. Northern blot analysis with a Runt-domain fragment as a probe showed that *Runx2* was only expressed in osteoblasts. Runx2 is an osteoblast-specific transcription activator. Ossification and osteoblasts formation are impaired in *Runx2* gene-deficient neonatal mice (*Runx2*−/−), as shown by X-ray and histological analysis. Mild osteodysplasty was identified in *Runx2* heterozygous knockout mice (*Runx2* +/−) with hypoplasia of the clavicle, delayed development of membranous bone development and persistent nonunion of the anterior and posterior fontanelles because of delayed skull ossification, which resembled human CCD syndrome^6^. Osteocalcin (OCN) is an osteoblast specific protein, regarded as a hallmark of osteoblast cells differentiation and maturation. The cis-element in the OCN gene promoter is OES2, which has an identical sequence to the Runt-binding site of *Runx2*^7^. The Runx2 binding site was also found in the promoter of bone sialoprotein (BSP), osteopontin (OPN) and COL I genes. Thus, Runx2 regulates the expression of osteoblast-specific genes.

In our study, we hypothesized that Runx2 enhances the osteogenic differentiation of ADSCs and the tendon-to-bone healing in ACL reconstruction. Herein, we found that ADSCs overexpressing Runx2 induced osteogenic differentiation *in vitro* and extensively regenerated new bone tissue at the transplantation site. In a rabbit ACL reconstruction model, with a slow-release fibrin glue matrix to deliver Runx2-ADSCs, we demonstrated that this material accelerated tendon-to-bone integration early after ACL reconstruction.

## Results

### Characteristics of ADSCs

**Figure 1:**
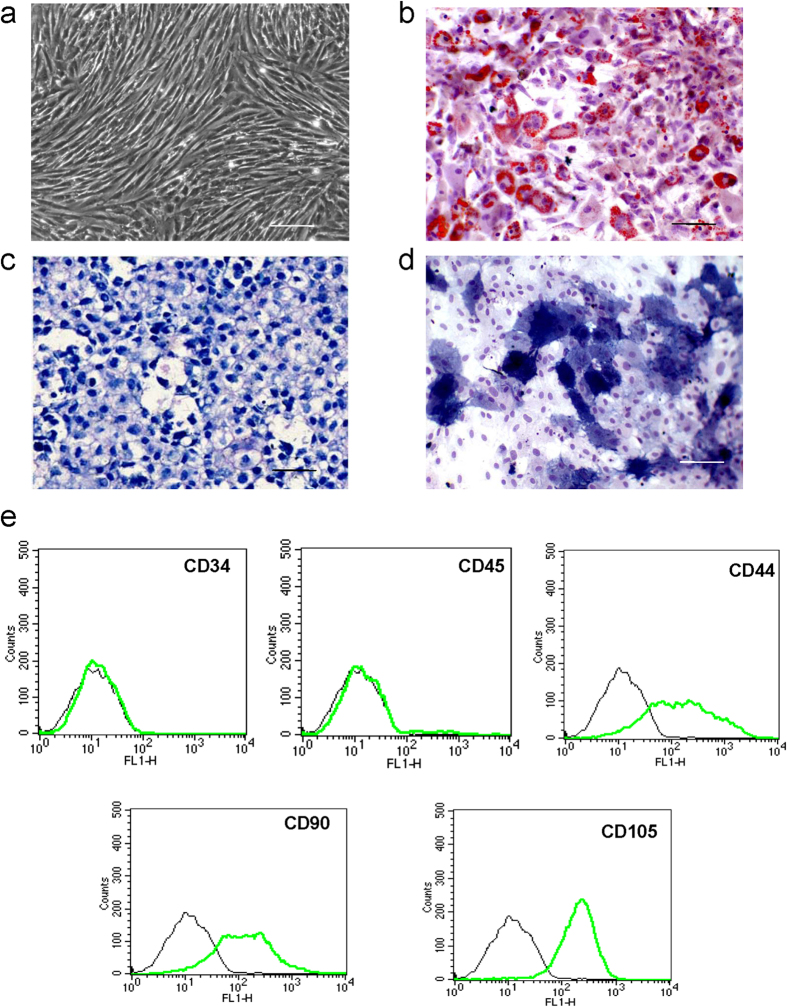
Identification of ADSCs by multi-lineage differentiation and cell surface markers. (**a**) Passage 3 ADSCs show fibroblast-like morphology (original magnification 100×). (**b**) Oil red stain shows lipid droplets 1 week after the induction of adipogenic differentiation. (**c**) ADSCs pellets were stained positively with toluidine blue after induction of chondrogenic differentiation at 14 days. (**d**) Alkaline phosphatase was detected in the cytoplasm 14 days after induction of osteogenic differentiation. The scale bar is 100 μm. (**e**) Specific cell surface markers were detected by flow cytometry. The ADSCs were negative for the hematopoietic lineage markers CD34 and CD45. The fractions of CD44-, CD90-, and CD105-positive cells were 67.63%, 68.13% and 92.43%, respectively, indicating their mesenchymal origin. The experiments were performed three times, and representative images are shown. Values are the mean ± SEM.

Approximately 2 × 10^6^ ADSCs were harvested from the inguinal groove adipose tissue. A few cells showed spontaneous adipogenesis in primary culture and were removed with passage. The cells in the third passage were almost all fibroblast-like cells (Fig. 1a). The doubling time of the cells was 3 days, reaching saturation in 4.5 days. Intracellular lipid droplets were observed by oil-red staining in ADSCs after adipogenic induction (Fig. 1b). ADSCs pellets were cultured in standard chondrogenic differentiation medium for 14 days, after which they were stained with toluidine blue to indicate the secretion of proteoglycan (Fig. 1c). Alkaline phosphatase (ALP) was detected in the cytoplasm after induction of osteogenesis (Fig. 1d), demonstrating the multipotency of the ADSCs. Specific cell surface markers were detected by flow cytometry: CD34 and CD45 were negative, CD44, CD90 and CD105 were positive (Fig. 1e).

### Adenoviral overexpression of Runx2 in ADSCs

**Figure 2:**
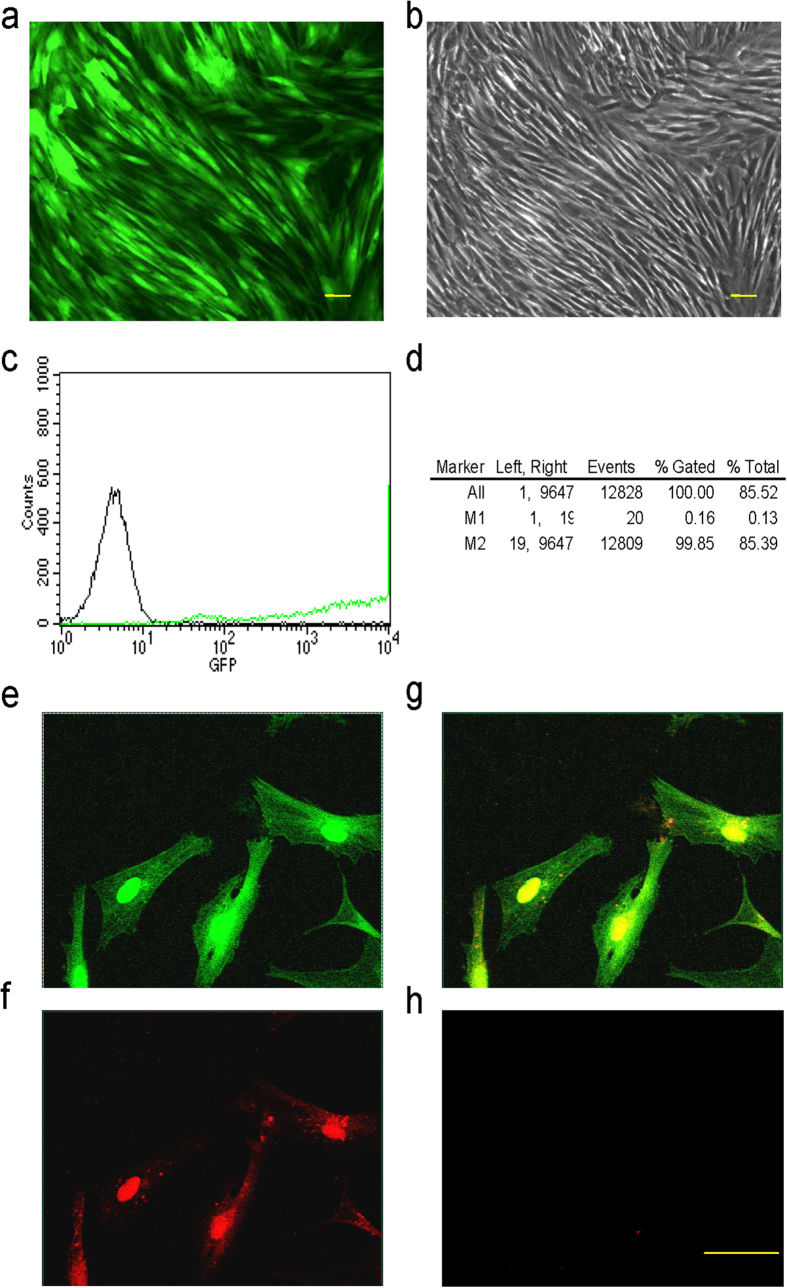
Infection of Ad-Runx2 (co-expression with EGFP) and expression of Runx2 in ADSCs. (**a,b**) Runx2 expression (green) in Ad-Runx2-infected ADSCs at 48 h post-infection was examined by fluorescence microscopy (**a**) and control cell morphology by light microscopy (**b**). (**c,d**) Ad-Runx2 transduction efficiency was measured by flow cytometry at 48 h post-infection (**c**) and using non-transduced ADSCs as controls for autofluorescence (**d**). (**e–h**) Immunofluorescence staining showed nuclear expression of Runx2 in Ad-Runx2-ADSCs at 48 h post-infection. EGFP (green) is expressed in the nucleus and cytoplasm (**e**), and Runx2 expression assayed by immuno-histology (red) is confined to the nucleus in infected cells (**f**). A merged image of (**e**,**f**) is shown in (**g**). Non-transduced cells were used as a negative control (**h**). The experiments were performed three times and representative images are shown. The scale bar is 20 μm.

ADSCs infected with Runx2 adenovirus (Ad-Runx2) (with co-expression of EGFP) were analyzed for EGFP expression by fluorescence microscopy and flow cytometry. High levels of EGFP were detected 48 h post-transduction using fluorescence microscopy (Fig. 2a,b). The infection efficiency of Ad-Runx2 in ADSCs was 99.69%, as indicated by flow cytometric detection of the EGFP marker 48 h post-infection. Non-infected cells were used as a control (Fig. 2c,d). Immunofluorescence staining showed nuclear expression of Runx2 in Runx2-ADSCs (ADSCs infected with Ad-Runx2). EGFP (green) was expressed in both the nucleus and the cytoplasm (Fig. 2e), while Runx2 expression (red) was confined to the nucleus in transduced cells (Fig. 2f). Figure 2g is the merged image of Fig. 2e,f. In the non-infected cells, no Runx2 was detected (Fig. 2h).

### Expression of osteogenic and adipogenic genes in ADSCs infected with Ad-Runx2

**Figure 3:**
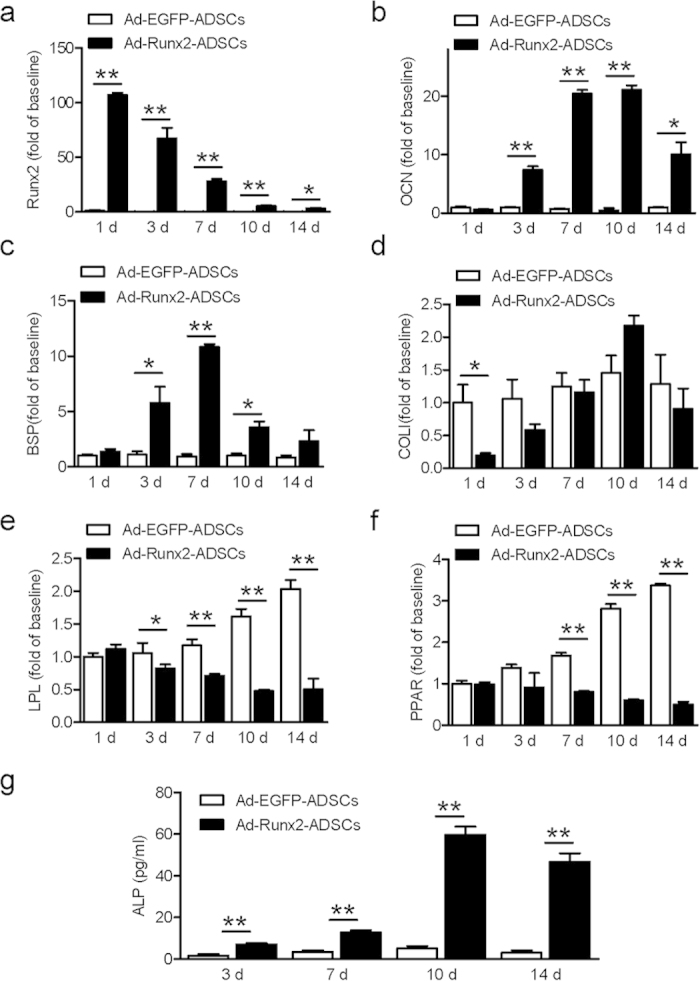
Expression of osteogenic and adipogenic genes in ADSCs infected with Ad-Runx2. (**a**) *Runx2* mRNA was detected by real-time RT-PCR in ADSCs infected with Ad-EGFP or Ad-Runx2 at 1, 3, 7, 10 and 14 days post-infection. (**b–d**) The expression of specific osteogenic genes, including OCN (**b**), BSP (**c**) and COLI (**d**) in ADSCs infected with Ad-EGFP or Ad-Runx2 at 1, 3, 7, 10 and 14 days post-infection, as assayed by real-time PCR. (**e**,**f**) The expressions of the specific adipogenic markers LPL (**e**) and PPARγ (**f**) mRNA levels by real-time RT-PCR in ADSCs infected with Ad-EGFP or Ad-Runx2 at 1, 3, 7, 10 and 14 days post-infection. (**g**) Analysis of ALP activity in the lysates of ADSCs infected with Ad-Runx2 or Ad-EGFP in at 2 3, 7, 10 and 14 days post-infection. ALP activity in Runx2-infected cells was elevated at day 3, continued to increase with a peak at 10 days, and then gradually declined, but remained at a high level for the duration of the experiment. The intrinsic cellular ALP activity in Ad-EGFP-infected cells remained unchanged. The experiments were performed three times, and representative images are shown. Values are mean ± SEM, **P < 0.01, *P < 0.05 *vs.* Ad-EGFP-ADSCs.

Using real-time RT-PCR, *Runx2* mRNA was detected in ADSCs infected with Ad-Runx2 at 1, 3, 7, 10 and 14 days post-infection, but not in Ad-EGFP-infected ADSCs (Fig. 3a). Upregulated mRNA expression of osteogenic genes, including OCN (Fig. 3b), BSP (Fig. 3c) and COLI (Fig. 3d) was observed in ADSCs infected with Ad-Runx2. The expression gradually increased and peaked at day 7 or day 10 post-infection, but not in ADSCs infected with Ad-EGFP. However, the expression of lipoprotein lipase (LPL) (Fig. 3e) and peroxisome proliferator activated receptor γ (PPARγ) (Fig. 3f) decreased dramatically in Ad-Runx2 infected ADSCs compared with the Ad-EGFP group. These results suggested that osteoblast differentiation was triggered by Runx2 and simultaneously, adipogenic differentiation was inhibited.

### Alkaline phosphatase activity in ADSCs infected with Ad-Runx2

ALP activity gradually increased with time in ADSCs infected with Ad-Runx2, peaked at day 10 post-infection and remained at a high level until day 14. The endogenous ALP activity in Ad-EGFP-infected ADSCs remained unchanged (Fig. 3g).

### Promotion of ectopic bone formation by Ad-Runx2-infected ADSCs *in vivo*

**Figure 4:**
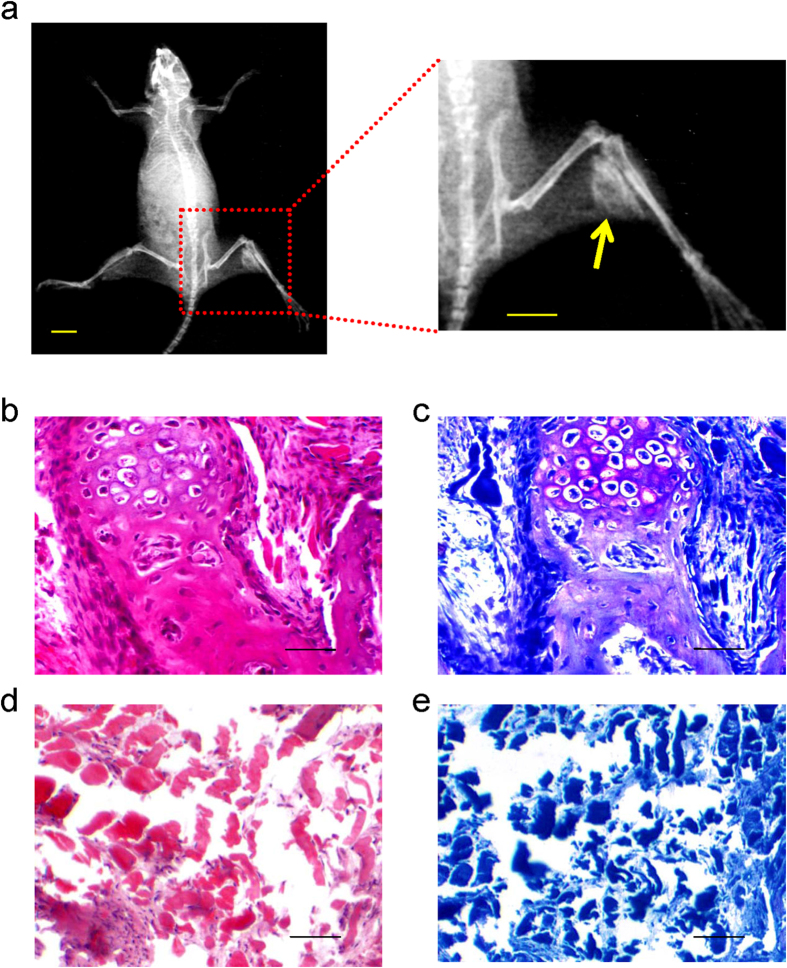
Ad-Runx2-infected ADSCs promote ectopic bone formation *in vivo*. (**a**) Bone formation was detected by X-rays in the muscle of right lower limb, 8 weeks after intramuscular injection of Ad-Runx2-modified ADSCs into the right lower limb of nude mice, but not in the left control limb injected with ADSCs infected with Ad-EGFP (scale bar is 10 mm). (**b–e**) Histological analyses of *in vivo* osteogenesis by Ad-Runx2-modified ADSCs. Note that significant amounts of cartilage and bone formation were observed with hematoxylin & eosin (**b**) and toluidine blue staining (**c**) in the Ad-Runx2-ADSCs group. The Ad-EGFP-ADSCs group contained muscle and fibrous tissue, with no evidence of bone formation (**d,e**). Representative images from six replications are shown.

A series of experiments was performed to examine the osteogenic activity of Ad-*Runx2*-infected ADSCs *in vivo*. We injected ADSCs infected with Ad-EGFP or Ad-Runx2 into the limbs of 2-week-old nude mice, as described in the Methods. 8 weeks after injection, we assessed the bone formation by X-rays and histological analysis. The X-ray images showed high-density signals in the right limb injected with Ad-Runx2-ADSCs, but not in the other limb with Ad-EGFP-ADSCs (Fig. 4a). Significant amounts of cartilage and bone formation were observed using hematoxylin & eosin and toluidine blue staining in the Ad-Runx2-ADSCs group (Fig. 4b,c). The control group contained muscle and fibrous tissue, with no evidence of bone formation (Fig. 4d,e). These data indicated that Ad-Runx2-infected ADSCs caused dramatic increases in osteogenic activity relative to control Ad-EGFP-ADSCs.

### Detection of bone tunnels by CT 3D reconstruction after ACL reconstruction surgery

**Figure 5:**
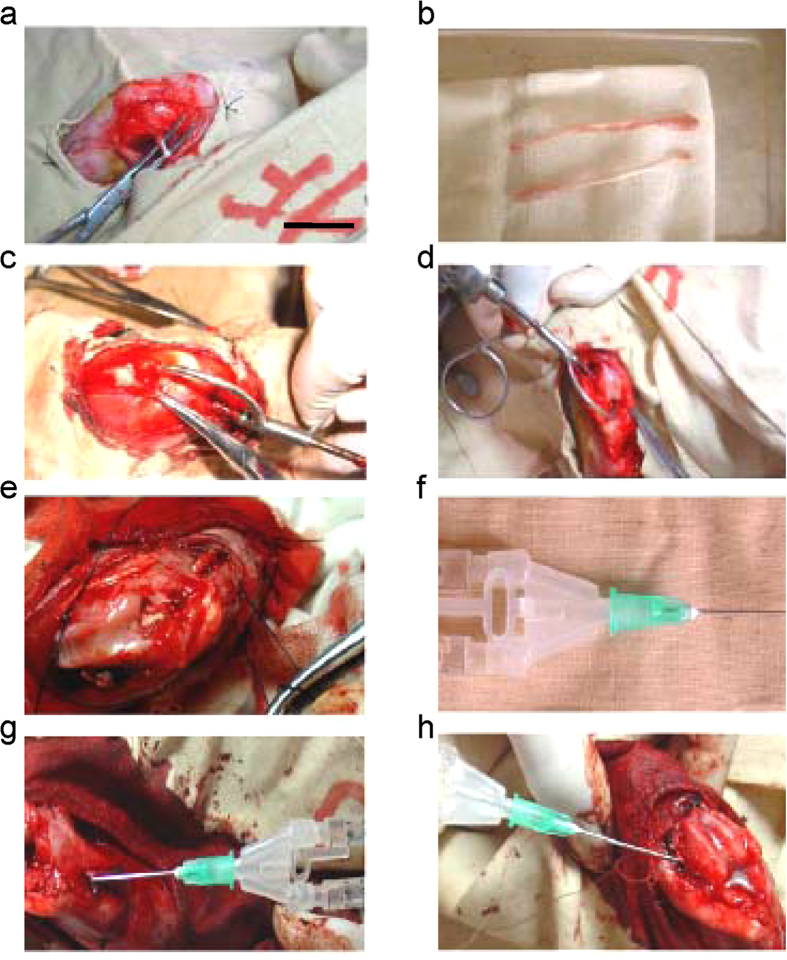
Surgical procedures for ACL reconstruction in rabbit models treated with Ad-Runx2-ADSCs, fibrin glue or RNAi Runx2. (**a,b**) The semitendinosus tendon was harvested. (**c,d**) Drilled tunnels (1.5 mm) were created through the femur (**c**) and tibia (**d**) at the insertion of the native ACL. (**e**) The graft was passed through the bone tunnels to replace the ACL. (**f**) Using a special needle equipped with fibrin glue, we mixed 0.2 ml of the fibrin glue with 1 × 10^6^ Runx2-ADSCs. (**g,h**) Injection around the periphery of the bone tunnel of the femur (**g**) and tibia (**h**) surrounding the tendon graft. Representative images from 10 replications are shown.

To confirm whether Runx2-ADSCs promoted the healing of the tendon-to-bone, we performed ACL reconstruction surgery in rabbits treated with fibrin glue, Ad-Runx2-ADSCs in fibrin glue or an RNA interference construct for Runx2 (RNAi Runx2; by injection of adenovirus overexpressing the Runx2 siRNA). The surgical procedures are shown in Fig. 5 and described in detail in the Methods.

**Figure 6:**
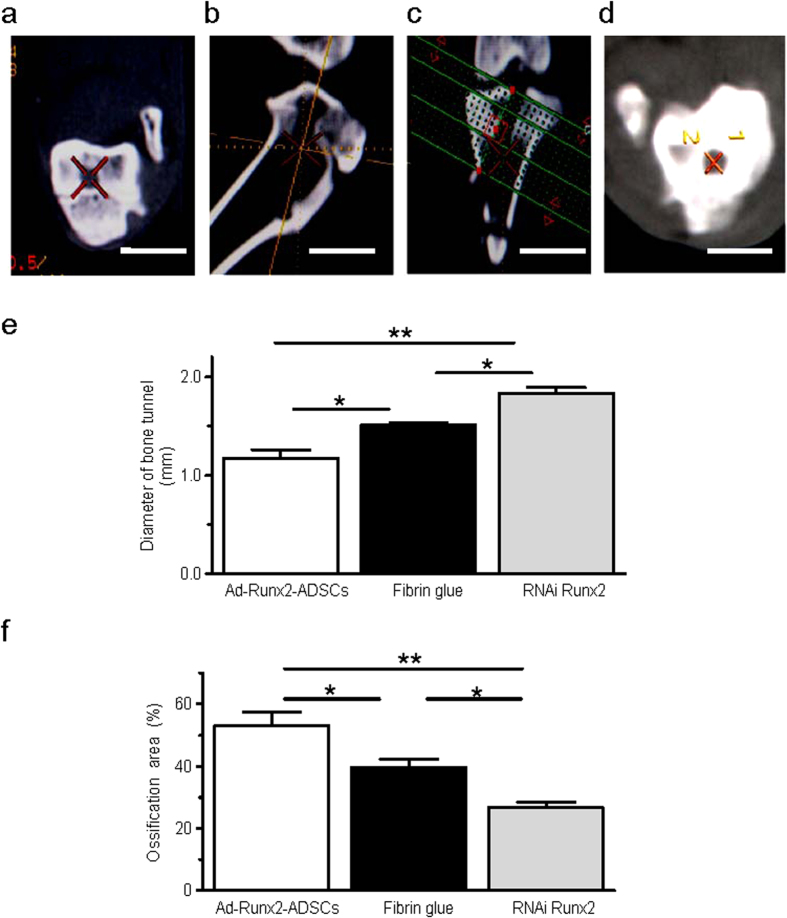
Evaluation of the width of bone tunnels by CT 3D reconstruction. (**a**–**d**) 12 weeks after surgery, the CT images were acquired to measure the CSA (cross sectional area) of the bone tunnels. First, we located the bone tunnel in cross sections (**a**). Then, using CT 3D reconstruction, the corresponding sagittal (**b**) and coronal (**c**) images were obtained to ensure that the orientation was perpendicular to the long axis of the bone tunnel. Based on the orientation, a series of cross sections were created, resulting in 1.0 mm-thick slices with no interslice gap (**d**). (**e**,**f**) Average diameters of bone tunnels (**e**) and the ossification areas (**f**) were analyzed from the CT 3D reconstruction of the joint after ACL reconstruction surgery treated with Ad-Runx2-ADSCs, fibrin glue or RNAi Runx2. Values are mean ± SEM, n = 10, *P < 0.05, **P < 0.01 as indicated.

After locating the cross-section perpendicular to the bone tunnel following CT 3D reconstruction of the knee, the average diameter of the bone tunnels in 12-week-old specimens were measured and compared (Fig. 6a–d). The bone tunnel diameters of the different groups were: Ad-Runx2-ADSCs group, 1.171 ± 0.245 mm; fibrin glue group, 1.506 ± 0.073 mm; RNAi Runx2 group, 1.831 ± 0.196 mm (Fig. 6e). The diameter of the bone tunnel in the animal model was 2.5 ± 0.532 mm. The areas of ossification in the tendon-to-bone were: Ad-Runx2-ADSCs group, 53.16 ± 7.56%; the fibrin glue group, 39.76 ± 4.37%; the RNAi Runx2 group, 26.76 ± 2.76% (Fig. 6f).

### Gross observation of the joint after ACL reconstruction surgery

**Figure 7:**
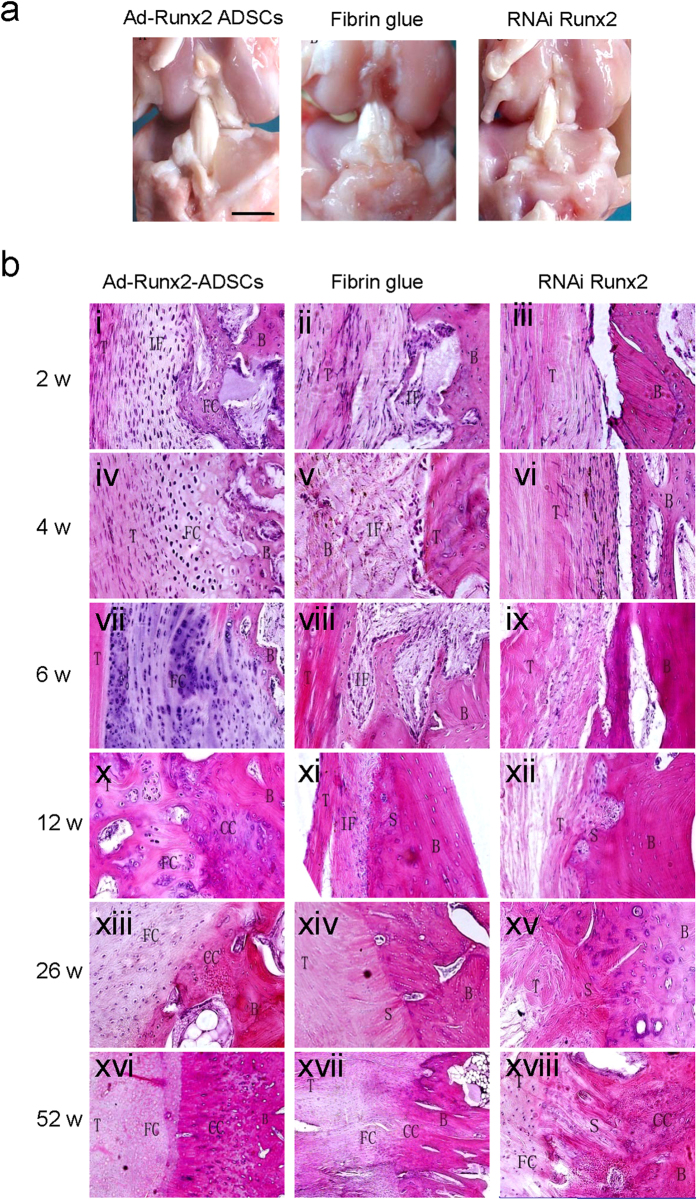
Gross observation and histological analysis of the joint after ACL reconstruction surgery on rabbits (**a**) Gross observation demonstrated no significant adverse effect of the treatments with Ad-Runx2-ADSCs, fibrin glue or RNAi Runx2 on the joint tissue. Note the lack of ossification or scar abundance at any of the intra-articular tissues, such as ligaments or menisci. (**b**) Histological photomicrographs of specimens post-surgery. Specimens were harvested and observed 2 (i, ii, iii), 4 (iv, v, vi), 6 (vii, viii, ix), 12 (x, xi, xii), 26 (xiii, xiv, xv), and 52 weeks (xvi, xvii, xviii) after surgery. Specimens were divided into three groups, includingAd-Runx2-ADSCs treated (i, iv, vii, x, xiii, xvi); fibrin glue treated (ii, v, viii, xi, xiv, xvii); and RNAi Runx2-treated (iii, vi, ix, xii, xv, xviii). T, tendon; IF, interface tissue; FC, fibrous cartilage; B, bone; CC, calcified cartilage; S, Sharpey fiber. Representative images from four replications are shown.

Gross observation demonstrated no significant adverse effect from the treatments with Ad-Runx2-ADSCs, fibrin glue or RNAiRunx2 on the joint tissues. No evidence of chondral degeneration or synovial inflammation was seen (Fig. 7a). Occasional heterotopic bone formation was observed at the extra-articular tunnel entrance in two knees in both the treated and the control groups (Fig. 7a). No ossification or scar abundance was seen at any intra-articular tissues, such as ligaments or menisci.

### Histological analysis of the joint after ACL reconstruction surgery

2 weeks after surgery, the tendon appeared viable with normal-appearing fibroblasts. There was a highly cellular, fibrovascular interface tissue (IF) between the tendon (T) and bone (B) in the Ad-Runx2-ADSCs-treated specimens. There was extensive formation of new bone trabeculae and cartilage in the tendon-bone interface (Fig. 7Bi). In the fibrin glue-treated specimens, healing occurred with the formation of a fibrovascular interface tissue between the tendon and bone. Occasional cartilage was formed in the interface zone (Fig. 7Bii). In the RNAiRunx2-treated specimens, a large gap existed between the tendon and bone, suggesting a disordered fibrous connective tissue. No evidence of foreign body giant cell response to the fibrin glue or any adverse effect associated with the articular cartilage of the tibia or femur was observed. Mild hyperplasia of the synovial membrane was seen in both treated and control limbs (Fig. 7Biii).

In Ad-Runx2-ADSCs-treated specimens 4 weeks after surgery, there was progressive formation of new fibrocartilage (FC) in the tendon-bone interface in close proximity to the tendon (Fig. 7Biv). Generally, more cartilage and fibrovascular tissue occurred in the tendon-bone interface compared with the 2-week specimens, and the cartilage tissue was more mature. The borderline between the tendon and bone was vague. The fibrin glue-treated specimens showed a persistent zone of fibrous interface tissue, which demonstrated increased extracellular matrix production and decreased cellularity and vascularity compared with the 2-week specimens. There was increased collagen fiber continuity between the new bone and the outer tendon, with a few chondrocyte-like cells appearing among the fibrous interface tissue (Fig. 7Bv). In the RNAiRunx2-treated specimens, the gap between bone and tendon was still present. Limited cell proliferation and fiber continuity occurred between the bone and the outer tendon. However, no evidence of new bone or cartilage formation was observed (Fig. 7Bvi).

In Ad-Runx2-ADSCs-treated specimens 6 weeks after surgery, graft necrosis showed regular fibrous structure with reduced tendon cells, significantly enhanced cartilage and bone formation, and remodeling around the bone tunnel. New fibrocartilage invaded the tendon with close apposition of new bone. The remnant fiber collagen was distributed sporadically in the new cartilage. There was progressive mineralization and maturation of the new bone lining in the bone tunnel. There appeared to be healing between the tendon and bone throughout the length of the bone tunnel (Fig. 7Bvii).

In the fibrin glue-treated specimens, the degree of healing varied between the tendon and bone. Most of the specimens demonstrated a persistent zone of wide interface between the tendon and bone with only loose fibrous tissue formation. There was increased collagen fiber continuity in the tendon-bone interface compared with the 4-week specimens. Tendon necrosis disappeared and instead, a few osteoblasts were observed in the tendon (Fig. 7Bviii). In the RNAiRunx2-treated specimens, the gap between bone and tendon had narrowed. The tendon-bone interface was filled with newly formed granulation tissue and fibrous connective tissue within new blood capillary and cells. The collagen fibers resembled Sharpey fibers (Fig. 7Bix).

In Ad-Runx2-ADSCs-treated specimens 12 weeks after surgery, the tendon-bone interface showed normal ACL attachment comprising tendon (T), fibrocartilage (FC), calcified cartilage (CC) and bone (B), arranged chaotically. The boundary between the tendon and bone was obscure. Progressive cartilage and bone matrix deposition in the tendon part of the bone tunnel was seen, along with newly formed trabecula and cavitas medullaris. The middle and distant articular parts of the bone tunnel were already filled with new bone (Fig. 7Bx). In the fibrin glue-treated specimens, the proliferated cells invaded the tendon in some of the control and treated specimens, with no obvious difference between groups. More compact Sharpey-like fibers were observed, linking the tendon with the bone directly at the tendon-bone interface. Only cartilage-like cells lined up along the interface (Fig. 7Bxi). In the RNAi Runx2-treated specimens, the gel between the tendon and bone disappeared and was replaced with a fibrovascular tissue comprising new cells and blood vessels. No evidence of cartilage or bone formation was observed, and no new chondrocytes or osteocytes were found in the interface (Fig. 7Bxii).

In Ad-Runx2-ADSCs-treated specimens 26 weeks after surgery, the tendon to bone interface was similar to a typical direct insertion of a normal ACL, with four zones: tendon, fibrocartilage, calcified cartilage and bone, organized with clear boundaries. The tide line between the fibrocartilage zone and the calcified cartilage zone was not very clear (Fig. 7Bxiii). In the fibrin glue-treated specimens, the tendon-bone interface was seen as an indirect insertion. Regular Sharpey-like fibers were arranged more compactly across the tendon-to-bone interface, with a large amount of newly formed fibrocartilage in the juncture (Fig. 7Bxiv). In the RNAi Runx2-treated specimens, a layer of cellular, fibrous tissue was formed between the tendon and the bone along the length of the bone tunnel. Collagen fiber continuity was established between the tendon and bone, but with no evidence of cartilage islands in the interface zone (Fig. 7Bxv).

Ad-Runx2-ADSCs-treated specimens 52 weeks after surgery showed a typical direct insertion of a normal ACL. The tide line between the fibrocartilage zone and calcified cartilage zone was clear (Fig. 7Bxvi). The fibrin glue-treated specimens showed four zones: tendon, fibrocartilage, calcified cartilage and bone, in the tendon-bone interface, but they were not organized uniformly. No tide line was found (Fig. 7Bxvii). The RNAiRunx2-treated specimens showed islands of fibrocartilage and calcified cartilage with disorganized collagen fibers between the tendon and bone. No direct insertion or tide line was apparent (Fig. 7Bxviii).

### Biomechanical testing of the joint after ACL reconstruction surgery

**Figure 8:**
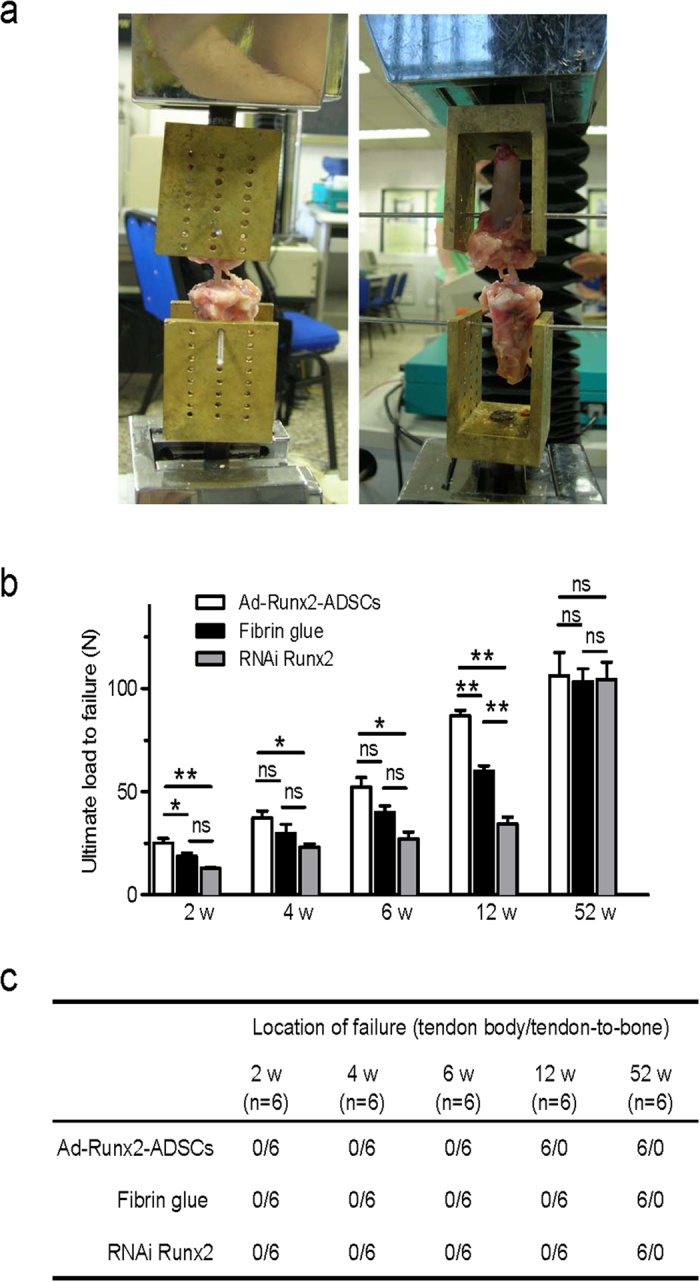
Biomechanical testing of the joint after ACL reconstruction surgery. (**a**) Images taken during the biomechanical analysis of the rabbits joints. The femur-ACL graft-tibia complexes were fixed in specially designed clamps, allowing tensile loading along the axis of the graft in a material testing machine. (**b,c**) The average ultimate load to failure (**b**) and failure location (**c**) of the graft in the Runx2-ADSCs, fibrin glue and RNAi Runx2 specimens at 2, 4, 6, 12 and 52weeks after surgery. Values are mean ± SEM, n = 6. *P < 0.05, **P < 0.01. ns, not significant.

In 2-, 4- and 6-week specimens, biomechanical testing was performed in a mechanical testing machine (Fig. 8a). The results demonstrated that the average maximum load at pullout was increased in the Ad-Runx2-ADSCs-treated specimens with time. The pullout strength in the Ad-Runx2-ADSCs-treated specimens was significantly greater than the control specimens (fibrin glue and RNAi Runx2) at 2, 4 and 6 weeks after surgery (Fig. 8b). The location of the failure of the grafts in the 3 groups was all at the tendon-to-bone (Fig. 8c).

Specimens harvested at 12 weeks after surgery for mechanical testing showed that the breakage site of the graft from the Ad-Runx2-ADSCs group was different from that of the other two groups (Fig. 8c). In the Ad-Runx2-ADSCs group, the tendons were torn in the middle of the tendon body. However, in the two control groups, the tendons were pulled out from the bone tunnel, indicating that the graft treated with Ad-Runx2-ADSCs was already incorporated successfully into the bone tunnel at 12 weeks after surgery.

There was no significant difference in ultimate load to failure between the groups at 52 weeks (Fig. 8b). There was no difference among Ad-Runx2-ADSCs, fibrin glue and siRNA Runx2 in terms of the failure sites. All specimens showed mid-substance failure, with significantly higher failure loads (Fig. 8c). The lack of difference indicated that Ad-Runx2-ADSCs promoted integration of the tendon to bone in the early healing period.

## Discussion

### The effect of Runx2 on osteogenesis is direct and efficient

Bone morphogenetic protein (BMP) is a member of transforming growth factor-β (TGF-β) family, with more than 10 members involved in osteoblast differentiation and bone formation regulation^8^. BMPs have multiple roles in the regulation of osteogenesis, which can be interfered with negative feedback of downstream factors. Runx2 is a transcription factor involved in osteogenesis, which is mainly mediated by BMPs, directly regulating the expression of osteoblast-specific proteins such as OCN and BSP, and the deposition of calcium^9^. In the current study, a remarkable time-dependent upregulation of the expression of OCN, BSP, and COL I was induced in ADSCs by overexpression of Runx2. In our *in vivo* study, Runx2-ADSCs were injected intramuscularly into the right lower limb, resulting in the formation of cartilage, bone, and bone marrow cavity 8 weeks later, which indicated the high efficacy of Runx2 for *in vivo* osteogenic induction in the non-osteogenic environment of ADSCs differentiation into bone tissues. In addition, Ad-Runx2-ADSCs gel was injected into tendon bone insertion of an ACL reconstruction model, resulting in new bone tissue formation at the junction between the tendon and bone 2 weeks later. A large number of chondrocytes were found around the tendon-bone insertion 6 weeks later, which clearly demonstrated the osteogenic induction potency of Runx2.

### Runx2 is more accurate in osteogenic induction and reduces the risk of heterotopic ossification

Runx2, which was expressed and functioned only in the transfected target cells without spread or interference with the adjacent host cells, is different from the secreted BMPs. Therefore, Runx2 is considered safer and more specific when used clinically. In our study, gross observation demonstrated no heterotopic ossification beyond the joint, intra-articular bone hyperplasia, or ossification of ligament and meniscus, indicating that Runx2, as a nucleic transcription factor, was more specifically localized than the secreted proteins. Therefore, Runx2 is more appropriate for healing of the tendon bone insertion after ACL reconstruction, which is connected to the articular cavity.

### Runx2 modulates the dynamic balance between various adipogenic and osteoplastic factors

One of the objectives of the current study was to test whether Runx2 inhibited the expression of LPL and PPARγ in ADSCs, both of which are key regulators of adipogenic differentiation and promote the differentiation of mesenchymal stem cells into adipocytes. Overexpression of Runx2 prevented the differentiation of ADSCs into adipocytes, although the mechanism remains to be determined. Our findings suggested that Runx2 modulates the dynamic balance between various adipogenic and osteoplastic factors, which is of great significance for the treatment of osteoporosis and bone defects.

### Runx2 modified-ADSCs promotes the healing of the tendon bone insertion at an early stage of ACL reconstruction

One of the key transplantation characteristics of the ACL is to create a secure insertion within the bone, and ensure the integrity of anatomical structure and normal physiological function of the reconstructed ligament. Normally, the ACL is inserted directly, characterized by fusion of the ligament with osteochondral tissue comprising tendon tissue, cartilage layer, calcified cartilage and bone tissue. Direct insertion is helpful for the transplant growth and remodeling, with reduced stretch load on the ligament: the transitional structure enables gradual increase in stiffness and distribution of the tension gradient from the ligament to the bone^10^. Indirect insertion is also known as fiber insertion, with the tendon fused to the bone at a certain angle. The connective tissue between the transplanted tendon and the bone tunnel is the collagen fiber (Sharpey fiber)^11^. Our findings indicated that Ad-Runx2-ADSCs treatment restored normal insertion of the ACL 12 weeks after surgery, which was much faster than previously reported^12^. The *de novo* cartilage was largely formed around the bone tunnel wall 2 weeks after surgery in the Ad-Runx2-ADSCs group, accompanied with osteoplastic activity and bone matrix formation. The vascularized granulation tissue appeared around the tendon bone, loosely connected with the bone tunnel wall to form primary direct insertion at 12 weeks after surgery. The typical direct insertion was characterized by four clearly demarcated layers of structure, including the tendon, fibrocartilage, calcified cartilage and bone. Our findings showed that Runx2-ADSCs directly promoted tendon-bone insertion remodeling and promoted successful outcome after ACL reconstruction with high efficiency and potency. However, in the conventional ACL reconstruction group, there was no obvious cartilage formation or ossification. The interface between the tendon and the bone was completely replaced by Sharpey-like dense fiber tissue, resembling indirect insertion. The indirect insertion usually occurred at a place with high shearing force, indicating that micro-stirring existed between the tendon and the bone^12^. We speculated that the indirect insertion by conventional ACL operation might result from slow *de novo* bone tissue formation and the micro-stirring of the tendon within the bone tunnel caused by postoperative activity of the animal. The tendon bone insertion was still chaotic at 52 weeks after surgery in the group subjected to Runx2 RNA interference. RNAi Runx2 inhibited *Runx2* expression in bone marrow stem cells, confirming that Runx2 played a vital role in the osteoblast differentiation and deposition of bone matrix.

### Maximum pullout load and biomechanical properties of reconstructed ACL

The tendon was pulled out from the bone tunnel at the early stage of reconstruction (2 to 6 weeks) from all the groups. At 6 weeks after operation, the pull-out load was very weak in the group treated with RNAiRunx2. The average pullout load was significantly higher in the Ad-Runx2-ADSCs treatment group compared with that of the other 2 groups. The biomechanical test at 12 weeks showed that the fracture occurred mainly in the ligament in the Ad-Runx2-ADSCs-treated group, while the avulsion occurred mainly in the junction of the tendon and bone in the RNAiRunx2 and fibrin glue groups. The pull-out load of the tendon bone insertion was significantly higher in theRunx2-ADSCs treatment group at various checkpoints compared with the control groups, suggesting that Runx2-ADSCs not only promoted the remodeling of the anatomical structure by ACL reconstruction, but also facilitated the restoration of physiological function of the tendon bone insertion. The maximum pull-out load of the reconstructed ACL was tested at 52 weeks by a biomechanical tensile experiment. No significant difference was observed in terms of maximum pull-out load in all three groups. In summary, Ad-Runx2-ADSCs treatment promoted tendon-bone healing at an early stage of ACL reconstruction, with significantly improved stability of the tendon bone insertion within 12 weeks. No adverse effects were associated with ADSCs as carriers for adenovirus treatment, or with the adenovirus associated RNAi or ACL reconstruction.

### CT 3D reconstruction as a non-invasive and accurate monitoring of ACL insertion healing

Imaging is an important tool to monitor the alterations associated with the bone tunnel position, changes and tendon bone healing after ACL reconstruction. The width of the bone tunnel is commonly measured by X-rays^13^. In general, using the bone tunnel ossification wall as the boundary, the diameter perpendicular to the longitudinal axis on the coronal and sagittal section were measured at the widest or self-defined position. Although X-ray measurement is simple, convenient and economic, its limitations include the dependence on ossification of bone tunnel wall, and the inaccuracy of bone tunnel inspection at an early stage of reconstruction (within 12 weeks). The accuracy of X-ray measurement is also affected by interference from variation in the posture of the knees during photography and magnification. Therefore, CT was used to evaluate the postoperative bone tunnel, which shows higher sensitivity for bone tunnel measurement during early stage of reconstruction^14^. CT ensures the accuracy and precision of the location of a series of bone tunnel cross sections by coronal, sagittal and axial inspection, without any postural variation. The widening of the bone tunnel indicated by X-rays was not related to joint stability after surgery, whereas the ossification area of the bone tunnel measured by CT was negatively correlated to KT2000^15^. CT 3D reconstruction was adopted in the current study to compare the diameter of the bone tunnel, as well as stability at the early stages of reconstruction. The effect of Runx2-ADSCs treatment on the healing of the tendon bone insertion was evaluated by imaging analysis. We found that *Runx2* overexpressing ADSCs increased new bone generation within the bone tunnel and promoted ligamentous ossification, which enabled transplant fixation within the bone tunnel and prevented bone tunnel enlargement.

In summary, multipotent ADSCs could differentiate selectively into osteoblasts following Runx2 overexpression. Runx2-modified ADSCs promoted new bone formation *in vivo* and *in vitro*, and enabled the healing of the tendon bone insertion after ACL reconstruction.

## Methods

### Animals

4-week-old male SD rats, 4-week-old male nude mice and 5-month-old male New Zealand white rabbits were purchased from the Beijing Animal Administration Center. All animal experimental protocols were approved by the Animal Care and Use Committee of Peking University and were in compliance with the “Guide for the Care and Use of Laboratory Animals”.

### Isolation and culture of ADSCs

ADSCs were harvested from the inguinal groove adipose tissue of 4-week-old male SD rats and 5-month-old male New Zealand white rabbits using a protocol described previously^16^. The isolated adipose tissue was then digested with 0.1% collagenase type I (Sigma) with intermittent shaking at 37 °C for 30 min. Enzyme activity was terminated by dilution with Dulbecco’s modified Eagle medium (DMEM), containing 10% fetal bovine serum (FBS) (HyClone, Logan, UT, USA). The floating adipocytes were separated from the stromal cell fraction by centrifugation (800 rpm) for 5 min. The pellets were filtered through a 200-μm nylon mesh to remove cellular debris and incubated overnight at 37 °C with 5% CO_2_ in culture medium (DMEM, 10% FBS, and 100 μl/ml penicillin/streptomycin). The primary cells were cultured for 4–5 days until they reached confluence and were defined as passage “0”. The cells were passaged at a ratio of 1:3. Cells between passages 3 and 10 were used in subsequent experiments.

### ADSC differentiation and identification

The specific cell surface antigen markers of ADSCs were examined by flow cytometry (FCM). The antibodies included those recognizing CD34 (ab187284), CD44 (ab157107), CD45 (ab10558), CD90 (ab92574) and CD105 (ab11414) (Abcam, HKSTP, N.T Hong Kong)^17^. A trilineage-induced differentiation experiment was also performed to identify the multiple differentiation potential of ADSCs, which include adipogenesis, chondrogenesis and osteogenesis. The ADSCs of passage three were used in all experiments. Briefly, the ADSCs were incubated in a six-well plate at a density of 1 × 10^5^ cells per well with Rat ADSC Adipogenic or Osteogenic Differentiation Medium (Cyagen Biosciences Inc, Sunnyvale, CA, USA) for adipogenesis or osteogenesis induction, respectively. The cells were examined for adipogenesis using oil-red O staining after 1 week of culture, or for osteogenesis through ALP staining after 2 weeks of culture. For chondrogenesis, pellet culture was performed. Briefly, the ADSCs were digested with trypsin, and resuspended in DMEM in a 15 ml polypropylene centrifuge tube. A total of 1 × 10^6^ cells/tube were washed with DMEM twice, centrifuged at 150 × *g* for 5 min, resuspended in 0.5 ml of Rat ADSC Chondrogenic Differentiation Medium (Cyagen Biosciences Inc., Sunnyvale, CA, USA), and centrifuged again. The pellet was incubated at the bottom of the tube with the supernatant at 37 °C in 5% CO_2_ for 24 h, and the tube was gently flicked to ensure the pellet was free-floating. The medium was changed every 2–3 days. After 3 weeks of incubation, the pellet was fixed in 4% paraformaldehyde and embedded in paraffin. Toluidine blue staining was then performed to assess the glycosaminoglycan formation in the extracellular matrix of the pellet.

### Construction of the adenovirus encoding Runx2

The AdEasy system (provided by Dr. T. He, Howard Hughes Medical Institute, Baltimore, MD) was used to construct recombinant adenovirus, as described previously^16^. Briefly, the pBS KS^-^plasmid containing the full-length *Runx2* cDNA (provided by Professor G. Karsenty, Department of Molecular and Human Genetics, Baylor College of Medicine, Huston, TX) was digested with Xho I and Xba I, resulting in a 3000-bp fragment containing the *Runx2* cDNA. The target fragment was inserted into the pAdTrack-CMV vector, which was linearized by digestion with Pme I, and subsequently co-transformed into *E*. *coli* BJ5183 with pAdEasy-1. Recombinants were selected for kanamycin resistance and recombination was confirmed by digestion with Pac I and EcoR I. Finally, the linearized recombinant plasmid was transfected into 293A cells using lipofectamine (Invitrogen, Carlsbad, CA, USA) for adenovirus packaging. Recombinant adenovirus carrying the *Runx2* gene (called Ad-Runx2) was propagated by reinfecting 293A cells and purified by CsCl gradient ultracentrifugation. Purified viral particles were stored in 10% glycerol/PBS until use at −80 °C at a concentration of 1 × 10^11^ plaque-forming units (pfu)/ml. A control recombinant adenovirus carrying the enhanced green fluorescence protein gene, Ad-EGFP, was constructed using the same method (1 × 10^11^ pfu/ml).

### Synthesis of siRNA for Runx2 and construction of Runx2-siRNA recombinant adenovirus

The target sequences in the *Runx2* mRNA were chosen according to Ambion’s siRNA design on-line tool. The specificity of all sequences was confirmed by BLAST searching. All siRNAs were synthesized with the silencer siRNA construction kit (Ambion, Austin, TX, USA), according to the manufacturer’s protocol. The negative control (scrambled siRNA) siRNA was purchased from Ambion. Transfection of siRNA was performed using siPORT_ Lipid (Ambion) according to the manufacturer’s protocol.

The Runx2-siRNA recombinant adenovirus was constructed with the pSilencer_adeno 1.0-CMV System (Ambion, Austin, TX, USA), according to the manufacturer’s protocol as reported previously^18^. Briefly, the synthesized siRNA was inserted into the shuttle vector 1.0 CMV at the XhoI and SpeI restriction enzyme sites. The resultant DNA and the adenoviral LacZ backbone were linearized with PacI and transfected in HEK293 cells using a calcium phosphate method for adenovirus packaging. The control recombinant adenovirus carrying the scrambled siRNA was constructed using the same method. The recombinant adenovirus was purified by CsCl ultracentrifugation. Purified viruses were dialyzed in phosphate-buffered saline (PBS) with 10% glycerol and stored at −70 °C until use.

### ALP activity

ALP activity was measured using an ALP assay kit (Zhong Shan Biochemical, Beijing, China). Briefly, 2 × 10^5^ ADSCs/well were seeded in 6-well plates (three wells per group). 24 hours later, cells were exposed to Ad-Runx2 (with an EGFP tag) or Ad-EGFP at an MOI (multiplicity of infection: pfu/cell) of 200, or to PBS. On day 3, 7, 10 and 14, the cells were harvested and dissociated in 0.1 M Tris (pH 7.4) containing 1% Triton X-100 and 5 mM MgCl_2_ by sonication. ALP activity was normalized against the protein concentration and expressed as U/g/min.

### Osteogenic activity of Ad-Runx2-modified ADSCs *in vivo*

After 24-hour culture, ADSCs infected with Ad-EGFP or Ad-Runx2, were harvested by centrifugation (800 rpm) for 5 min, and resuspended in fresh medium at 2 × 10^6^ cells/ml. 1 × 10^6^cells in 0.5 ml medium were mixed with fibrin glue (0.2 ml) (TISSEEL Kit, IMMUNO AG, Vienna, Austria) and injected into the hind limb muscles of nude mice (n = 10). The right limb was injected with Ad-Runx2-ADSCs, and the left was injected with Ad-EGFP-ADSCs as a control. Radiological and histological evaluations were conducted 8 weeks post-injection. All mice were allowed to move freely in their cages.

### Surgical procedures of ACL reconstruction

All animals were operated on under general anesthesia (20% ethyl carbamate), according to standard procedures reported previously^19^. An anteromedial incision was made and a medial parapatellar arthrotomy was used to expose and resect the native ACL. The semitendinosus tendon was harvested (Fig. 5a,b), and drill tunnels (1.5 mm) were created through the femur and tibia at the insertion site of the native ACL (Fig. 5c,d). The graft was passed through the bone tunnels to replace the ACL (Fig. 5e). The tunnels were irrigated with normal saline before application of the experimental agents. All the experimental animals were divided into the groups:

Group I (the Ad-Runx2-ADSCs group) (n = 10). A total of 1 × 10^6^ Ad-Runx2-ADSCs immobilized in 0.2 ml fibrin glue (Fig. 5f) was injected in the bone tunnel of femur (Fig. 5g) and tibia (Fig. 5h) on the right limb. Using a special needle equipped with fibrin glue (Fig. 5f), we mixed 0.2 ml of the fibrin glue with 1 × 10^6^ Runx2-ADSCs and then injected them around the periphery of each tunnel surrounding the tendon graft. The graft was slid back and forth to ensure even coating of the fibrin glue along the entire tunnel.

Group II (the fibrin glue-treated group) (n = 10). Only 0.2 ml fibrin glue without cells was injected in the bone tunnel as above.

Group III (RNAi Runx2 group) (n = 10). We injected 0.2 ml of recombinant adenovirus (1 × 10^11^ PFU/ml) for Runx2-siRNA (Ad-RNAi-Runx2) into the open medullary cavity on the inner surface of bone tunnel and the part of hamstring tendon in bone tunnel. The other surgical procedures were the same as mentioned above. Ten specimens from each group were harvested at 2, 4, 6, 12, 24 and 52 weeks postoperatively and evaluated using CT 3D-reconstruction, histological and biomechanical methods.

### Evaluation of the width of bone tunnel by CT 3D reconstruction

12 weeks after surgery, the CT images of 30 specimens (10 specimens from each group) were acquired to measure the CSA (cross sectional area) of the bone tunnel. CT images were acquired on GE volume speed after first locating the bone tunnel in the cross sections (Fig. 6a). Using CT 3D-reconstruction, the corresponding coronal and sagittal images were obtained to ensure that the orientation was perpendicular to the long axis of the bone tunnel (Fig. 6b,c). Base on the orientation, a series of cross sections were created, resulting in 1.0 mm-thick slices with no interslice gap (Fig. 6d). Images were obtained tangential to the coronal and sagittal orientation of the long axis of the bone tunnel. These series of cross sections were viewed on a Magic View Workstation (Siemens, Erlangen), and the CSA of the tunnel was measured digitally using a computer generated best-fit circle.

### Biomechanical analysis

6 rabbits were killed at 2, 4, 6, 12, 26 and 52 weeks after surgery and frozen at −80 °C until biomechanical analysis, as described previously^20^. The limbs were thawed and all soft tissue was removed except the grafted tendon. All scar tissue and sutures at the femoral tunnel exits were carefully removed. The femur-ACL graft-tibia complexes were fixed in specially designed clamps (Fig. 8a), allowing tensile loading along the axis of the graft in a mechanical testing machine. A preload of 5N was applied. After cyclic preconditioning of the constructs between elongation limits of 0 and 0.5 mm, a load-to-failure test was performed at an elongation rate of 500 mm/min. The maximal pullout or failure load was recorded. The data were analyzed statistically, with P < 0.05 as the level of significance. The site of graft failure (femoral tunnel, mid-substance, or tibial tunnel) was recorded.

### Statistical analysis

All data were expressed as mean ± SEM and represented at least three independent experiments. Data analysis was performed using PASW Statistics 18 software. After testing for homogeneity of variances, an ANOVA test was used to evaluate the results among the three groups; at the same time, Pairwise Post Hoc Tests were performed with a least significant difference (LSD) multiple comparisons procedure. A Student’s *t*-test was used to evaluate results between two groups. *P* < 0.05 was considered statistically significant.

## Additional Information

**How to cite this article**: Zhang, X. *et al.* Runx2-Modified Adipose-Derived Stem Cells Promote Tendon Graft Integration in Anterior Cruciate Ligament Reconstruction. *Sci. Rep.* **6**, 19073; doi: 10.1038/srep19073 (2016).
